# IMP: a pipeline for reproducible reference-independent integrated metagenomic and metatranscriptomic analyses

**DOI:** 10.1186/s13059-016-1116-8

**Published:** 2016-12-16

**Authors:** Shaman Narayanasamy, Yohan Jarosz, Emilie E. L. Muller, Anna Heintz-Buschart, Malte Herold, Anne Kaysen, Cédric C. Laczny, Nicolás Pinel, Patrick May, Paul Wilmes

**Affiliations:** 1 Luxembourg Centre for Systems Biomedicine, 7, avenue des Hauts-Fourneaux, Esch-sur-Alzette, L-4362 Luxembourg; 2 Present address: Department of Microbiology, Genomics and the Environment, UMR 7156 UNISTRA—CNRS, Université de Strasbourg, Strasbourg, France; 3 Present address: Saarland University, Building E2 1, Saarbrücken, 66123 Germany; 4 Institute of Systems Biology, 401 Terry Avenue North, Seattle, WA 98109 USA; 5 Present address: Universidad EAFIT, Carrera 49 No 7 sur 50, Medellín, Colombia

**Keywords:** Multi-omics data integration, Metagenomics, Metatranscriptomics, Microbial ecology, Microbiome, Reproducibility

## Abstract

Existing workflows for the analysis of multi-omic microbiome datasets are lab-specific and often result in sub-optimal data usage. Here we present IMP, a reproducible and modular pipeline for the integrated and reference-independent analysis of coupled metagenomic and metatranscriptomic data. IMP incorporates robust read preprocessing, iterative co-assembly, analyses of microbial community structure and function, automated binning, as well as genomic signature-based visualizations. The IMP-based data integration strategy enhances data usage, output volume, and output quality as demonstrated using relevant use-cases. Finally, IMP is encapsulated within a user-friendly implementation using Python and Docker. IMP is available at http://r3lab.uni.lu/web/imp/ (MIT license).

**Electronic supplementary material:**

The online version of this article (doi:10.1186/s13059-016-1116-8) contains supplementary material, which is available to authorized users.

## Background

Microbial communities are ubiquitous in nature and govern important processes related to human health and biotechnology [1, 2]. A significant fraction of naturally occurring microorganisms elude detection and investigation using classic microbiological methods due to their unculturability under standard laboratory conditions [3]. The issue of unculturability is largely circumvented through the direct application of high-resolution and high-throughput molecular measurements to samples collected in situ [4–6]. In particular, the application of high-throughput next-generation sequencing (NGS) of DNA extracted from microbial consortia yields metagenomic (MG) data which allow the study of microbial communities from the perspective of community structure and functional potential [4–6]. Beyond metagenomics, there is also a clear need to obtain functional readouts in the form of other omics data. The sequencing of reverse transcribed RNA (cDNA) yields metatranscriptomic (MT) data, which provides information about gene expression and therefore allows a more faithful assessment of community function [4–6]. Although both MG and MT data allow unprecedented insights into microbial consortia, the integration of such multi-omic data is necessary to more conclusively link genetic potential to actual phenotype in situ [4, 6]. Given the characteristics of microbial communities and the resulting omic data types, specialized workflows are required. For example, the common practice of subsampling collected samples prior to dedicated biomolecular extractions of DNA, RNA, etc. has been shown to inflate variation, thereby hampering the subsequent integration of the individual omic datasets [7, 8]. For this purpose, specialized wet-lab methods which allow the extraction of concomitant DNA, RNA, proteins, and metabolites from single, unique samples were developed to ensure that the generated data could be directly compared across the individual omic levels [7, 8]. Although standardized and reproducible wet-lab methods have been developed for integrated omics of microbial communities, corresponding bioinformatic analysis workflows have yet to be formalized.

Bioinformatic analysis methods for MG and MT NGS data can be broadly classified into reference-dependent or reference-independent (de novo) methods [5]. Reference-dependent methods are based on the alignment/mapping of sequencing reads onto isolate genomes, gene catalogs, or existing MG data. A major drawback of such methods is the large number of sequencing reads from uncultured species and/or divergent strains which are discarded during data analysis, thereby resulting in the loss of potentially useful information. For example, based on analyses of MG data from the human gut microbiome (arguably the best characterized microbial community in terms of culture-derived isolate genomes), approximately 43% of the data are typically not mappable to the available isolate genomes [9]. Conversely, reference-independent methodologies, such as approaches based on de novo assemblies, enable the retrieval of the actual genomes and/or potentially novel genes present in samples, thereby allowing more of the data to be mapped and exploited for analysis [4, 5, 10]. Furthermore, it has been demonstrated that the assembly of sequencing reads into longer contiguous sequences (contigs) greatly improves the taxonomic assignments and prediction of genes as opposed to their direct identification from short sequencing reads [11, 12]. Finally, de novo MG assemblies may be further leveraged by binning the data to resolve and retrieve population-level genomes, including those from hitherto undescribed taxa [13–21].

Given the advantages of reference-independent methods, a wide array of MG-specific assemblers such as IDBA-UD [22] and MEGAHIT [23] have been developed. Most MT data analyses involve reference-based [24–26] or MG-dependent analysis workflows [27–29]. A comparative study by Celaj et al. [12] demonstrated that reference-independent approaches for MT data analyses are also applicable using either specialized MT assemblers (e.g., IDBA-MT [12, 30]), MG assemblers (e.g., IDBA-UD [22, 30, 31] and MetaVelvet [12, 32]) or single-species transcriptome assemblers (e.g., Trinity [12, 33]). In all cases, the available assemblers are able to handle the uneven sequencing depths of MG and MT data. Although dedicated assembly methods have been developed for MG and MT data, formalized pipelines allowing the integrated use of both data types are not available yet.

Automated bioinformatic pipelines have so far been mainly developed for MG data. These include MOCAT [34] and MetAMOS [10], which incorporate the entire process of MG data analysis, ranging from preprocessing of sequencing reads, de novo assembly, and post-assembly analysis (read alignment, taxonomic classification, gene annotation, etc.). MOCAT has been used in large-scale studies such as those within the MetaHIT Consortium [35, 36], while MetAMOS is a flexible pipeline which allows customizable workflows [10]. Both pipelines use SOAPdenovo [37] as the default de novo assembler, performing single-length *k*mer-based assemblies which usually result in fragmented (low contiguity) assemblies with low gene coverage values [38].

Multi-omic analyses have already provided new insights into microbial community structure and function in various ecosystems. These include studies of the human gut microbiome [28, 39], aquatic microbial communities from the Amazon river [27], soil microbial communities [40, 41], production-scale biogas plants [29], hydrothermal vents [42], and microbial communities from biological wastewater treatment plants [43, 44]. These studies employed differing ways for analyzing the data, including reference-based approaches [27, 28, 42], MG assembly-based approaches [29, 40], MT assembly-based approaches [42], and integrated analyses of the meta-omic data [39, 42–44]. Although these studies clearly demonstrate the power of multi-omic analyses by providing deep insights into community structure and function, standardized and reproducible computational workflows for integrating and analyzing the multi-omic data have so far been unavailable. Importantly, such approaches are, however, required to compare results between different studies and systems of study.

Due to the absence of established tools/workflows to handle multi-omic datasets, most of the aforementioned studies utilized non-standardized, ad hoc analyses, mostly consisting of custom workflows, thereby creating a challenge in reproducing the analyses [10, 45–47]. Given that the lack of reproducible bioinformatic workflows is not limited to those used for the multi-omic analysis of microbial consortia [10, 45–47], several approaches have recently been developed with the explicit aim of enhancing software reproducibility. These include a wide range of tools for constructing bioinformatic workflows [48–50] as well as containerizing bioinformatic tools/pipelines using Docker [29, 46–48].

Here, we present IMP, the Integrated Meta-omic Pipeline, the first open source de novo assembly-based pipeline which performs standardized, automated, flexible, and reproducible large-scale integrated analysis of combined multi-omic (MG and MT) datasets. IMP incorporates robust read preprocessing, iterative co-assembly of metagenomic and metatranscriptomic data, analyses of microbial community structure and function, automated binning, as well as genomic signature-based visualizations. We demonstrate the functionalities of IMP by presenting the results obtained on an exemplary data set. IMP was evaluated using datasets from ten different microbial communities derived from three distinct environments as well as a simulated mock microbial community dataset. We compare the assembly and data integration measures of IMP against standard MG analysis strategies (reference-based and reference-independent) to demonstrate that IMP vastly improves overall data usage. Additionally, we benchmark our assembly procedure against available MG analysis pipelines to show that IMP consistently produces high-quality assemblies across all the processed datasets. Finally, we describe a number of particular use cases which highlight biological applications of the IMP workflow.

## Results

### Overview of the IMP implementation and workflow

**Fig. 1:**
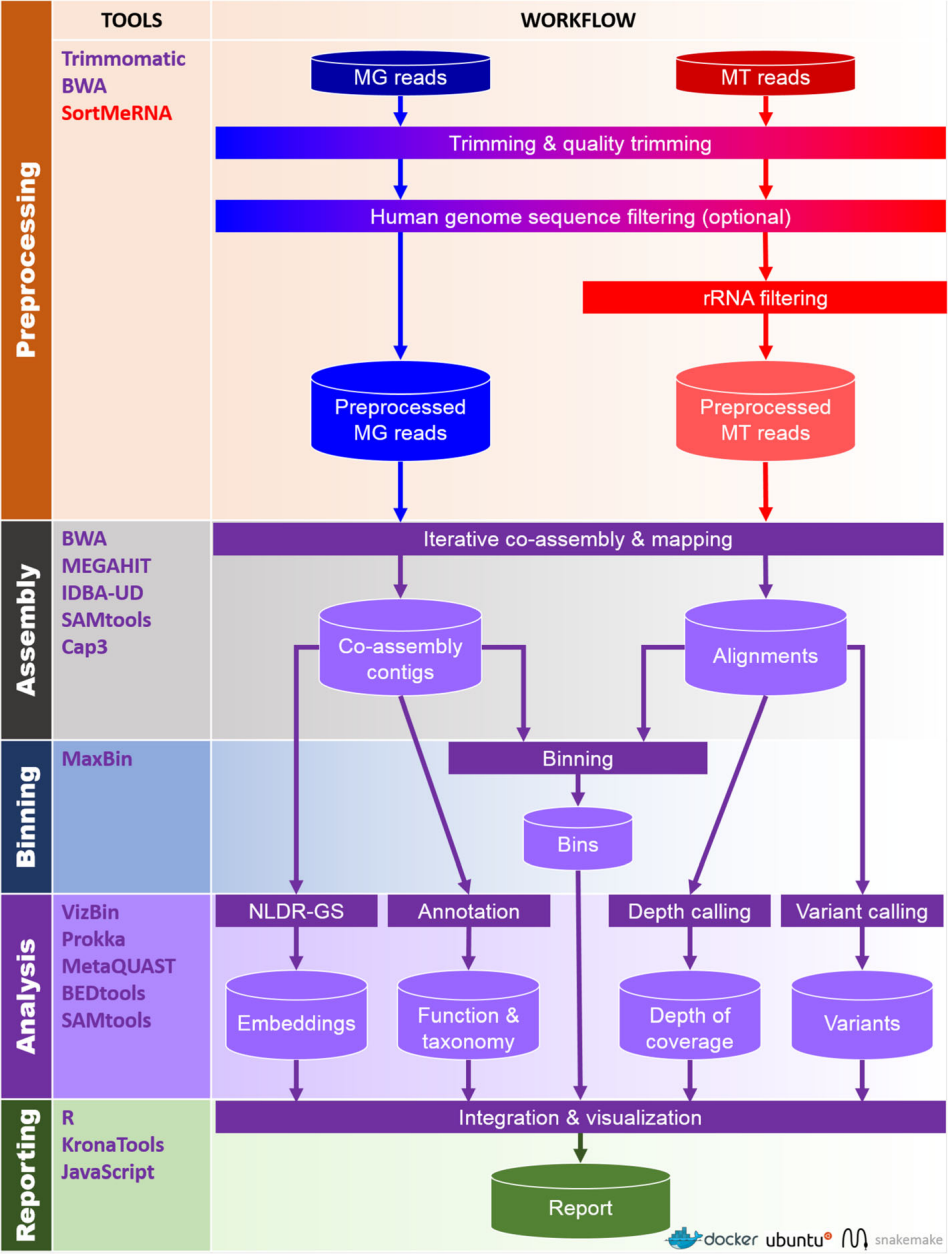
Schematic overview of the IMP pipeline. *Cylinders* represent input and output while *rectangles* represent processes. *Arrows* indicate the flow between input, processes, and output. *MG ﻿—* Metagenomic data, *MT —* Metatranscriptomic data, *rRNA ﻿—* ribosomal RNA, *NLDR-GS ﻿—* genomic signature non-linear dimensionality reduction. Processes, input, and output specific to MG and MT data are labeled in *blue* and *red*, respectively. Processes and output that involve usage of both MG and MT data are represented in *purple*. A detailed illustration of the “iterative co-assembly” is available in Additional file 1: Figure S1

IMP leverages Docker for reproducibility and deployment. The interfacing with Docker is facilitated through a user-friendly Python wrapper script (see the “Details of the IMP implementation and workflow” section). As such, Python and Docker are the only prerequisites for the pipeline, enabling an easy installation and execution process. Workflow implementation and automation is achieved using Snakemake [49, 51]. The IMP workflow can be broadly divided into five major parts: i) preprocessing, ii) assembly, iii) automated binning, iv) analysis, and v) reporting (Fig. 1).

The preprocessing and filtering of sequencing reads is essential for the removal of low quality bases/reads, and potentially unwanted sequences, prior to assembly and analysis. The input to IMP consists of MG and MT (the latter preferably depleted of ribosomal RNA prior to sequencing) paired-end reads in FASTQ format (section “Input data”). MG and MT reads are preprocessed independently of each other. This involves an initial quality control step (Fig. 1 and section “Trimming and quality filtering”) [52] followed by an optional screening for host/contaminant sequences, whereby the default screening is performed against the human genome while other host genome/contaminant sequences may also be used (Fig. 1 and section “Screening host or contaminant sequences”). In silico rRNA sequence depletion is exclusively applied to MT data (Fig. 1 and section “Ribosomal RNA filtering”).

The customized assembly procedure of IMP starts with an initial assembly of preprocessed MT reads to generate an initial set of MT contigs (Additional file 1: Figure S1). MT reads unmappable to the initial set of MT contigs undergo a second round of assembly. The process of assembling unused reads, i.e., MG or MT reads unmappable to the previously assembled contigs, is henceforth referred to as “iterative assembly”. The assembly of MT reads is performed, first as transcribed regions are covered much more deeply and evenly in MT data. The resulting MT-based contigs represent high-quality scaffolds for the subsequent co-assembly with MG data, overall leading to enhanced assemblies [43]. Therefore, the combined set of MT contigs from the initial and iterative MT assemblies are used to enhance the subsequent assembly with the MG data. MT data are assembled using the MEGAHIT de novo assembler using the appropriate option to prevent the merging of bubbles within the de Bruijn assembly graph [23, 36]. Subsequently, all preprocessed MT and MG reads, together with the generated MT contigs, are used as input to perform a first co-assembly, producing a first set of co-assembled contigs. The MG and MT reads unmappable to this first set of co-assembled contigs then undergo an additional iterative co-assembly step. IMP implements two assembler options for the de novo co-assembly step, namely IDBA-UD or MEGAHIT. The contigs resulting from the co-assembly procedure undergo a subsequent assembly refinement step by a contig-level assembly using the cap3 [53] de novo assembler. This aligns highly similar contigs against each other, thus reducing overall redundancy by collapsing shorter contigs into longer contigs and/or improving contiguity by extending contigs via overlapping contig ends (Additional file 1: Figure S1). This step produces the final set of contigs. Preprocessed MG and MT reads are then mapped back against the final contig set and the resulting alignment information is used in the various downstream analysis procedures (Fig. 1). In summary, IMP employs four measures for the de novo assembly of preprocessed MG and MT reads, including: i) iterative assemblies of unmappable reads, ii) use of MT contigs to scaffold the downstream assembly of MG data, iii) co-assembly of MG and MT data, and iv) assembly refinement by contig-level assembly. The entire de novo assembly procedure of IMP is henceforth referred to as the “IMP-based iterative co-assembly” (Additional file 1: Figure S1).

Contigs from the IMP-based iterative co-assembly undergo quality assessment as well as taxonomic annotation [54] followed by gene prediction and functional annotation [55] (Fig. 1 and section “Annotation and assembly quality assessment”). MaxBin 2.0 [20], an automated binning procedure (Fig. 1 and section “Automated binning”) which performs automated binning on assemblies produced from single datasets, was chosen as the de facto binning procedure in IMP. Experimental designs involving single coupled MG and MT datasets are currently the norm. However, IMP’s flexibility does not forego the implementation of multi-sample binning algorithms such as CONCOCT [16], MetaBAT [18], and canopy clustering [15] as experimental designs evolve in the future.

Non-linear dimensionality reduction of the contigs’ genomic signatures (Fig. 1 and section “Non-linear dimensionality reduction of genomic signatures”) is performed using the Barnes-Hut Stochastic Neighborhood Embedding (BH-SNE) algorithm allowing visualization of the data as two-dimensional scatter plots (henceforth referred to as VizBin maps [13, 56]). Further analysis steps include, but are not limited to, calculations of the contig- and gene-level depths of coverage (section “Depth of coverage”) as well as the calling of genomic variants (variant calling is performed using two distinct variant callers; section “Variant calling”). The information from these analyses are condensed and integrated into the generated VizBin maps to produce augmented visualizations (sections “Visualization and reporting”). These visualizations and various summaries of the output are compiled into a HTML report (examples of the HTML reports available via Zenodo [57]).

**Fig. 2:**
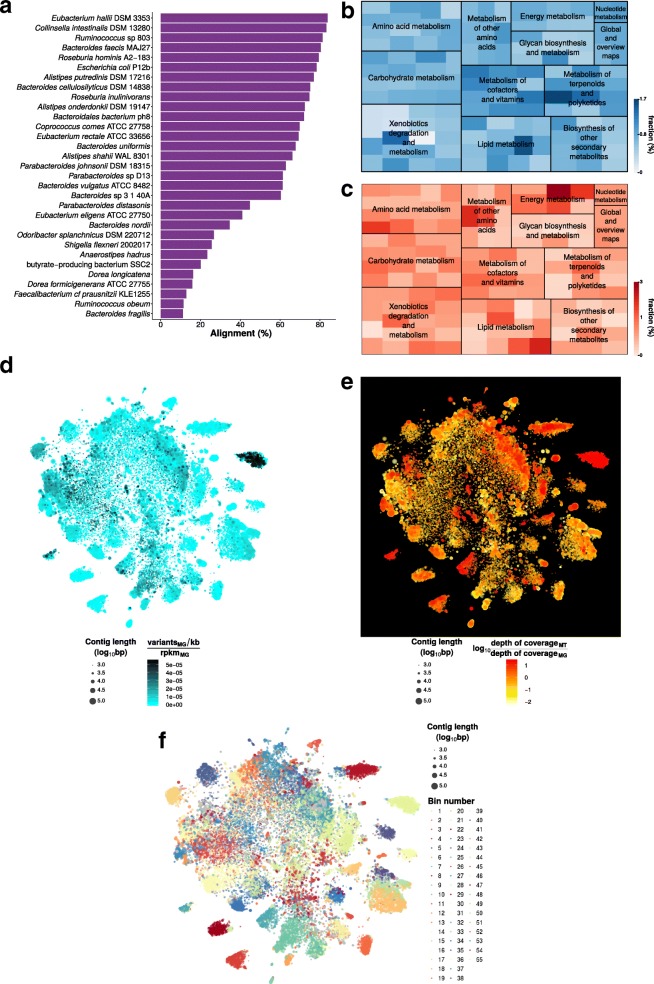
Example output from the IMP analysis of a human microbiome dataset (HF1). **a** Taxonomic overview based on the alignment of contigs to the most closely related genomes present in the NCBI genome database (see also HTML report S1 [57]). **a**, **b** Abundances of predicted genes (based on average depths of coverage) of various KEGG Ontology categories represented both at the MG (**b**) and MT (**c**) levels (see also Krona charts within HTML report S1). **d**–**f** Augmented VizBin maps of contigs ≥1 kb, representing contig-level MG variant densities (**d**), contig-level ratios of MT to MG average depth of coverage (**e**), and bins generated by the automated binning procedure (**f**). Please refer to the HTML reports [57] for additional examples

Exemplary output of IMP (using the default IDBA-UD assembler) based on a human fecal microbiome dataset is summarized in Fig. 2. The IMP output includes taxonomic (Fig. 2a) and functional (Fig. 2b, c) overviews. The representation of gene abundances at the MG and MT levels enables comparison of potential (Fig. 2b) and actual expression (Fig 2c) for specific functional gene categories (see Krona charts within HTML S1 [57]). IMP provides augmented VizBin maps [13, 56], including, for example, variant densities (Fig. 2d) as well as MT to MG depth of coverage ratios (Fig. 2e). These visualizations may aid users in highlighting subsets of contigs based on certain characteristics of interest, i.e., population heterogeneity/homogeneity, low/high transcriptional activity, etc. Although an automated binning method [20] is incorporated within IMP (Fig. 2f), the output is also compatible with and may be exported to other manual/interactive binning tools such as VizBin [56] and Anvi’o [17] for additional manual curation. Please refer to the HTML reports for additional examples [57].

The modular design (section “Automation and modularity”) and open source nature of IMP allow for customization of the pipeline to suit specific user-defined analysis requirements (section “Customization and further development”). As an additional feature, IMP also allows single-omic MG or MT analyses (section “Details of the IMP implementation and workflow”). Detailed parameters for the processes implemented in IMP are described in the section “Details of the IMP implementation and workflow” and examples of detailed workflow schematics are provided within the HTML reports [57].

### Assessment and benchmarking

IMP was applied to ten published coupled MG and MT datasets, derived from three types of microbial systems, including five human fecal microbiome samples (HF1, HF2, HF3, HF4, HF5) [28], four wastewater sludge microbial communities (WW1, WW2, WW3, WW4) [43, 44], and one microbial community from a production-scale biogas (BG) plant [29]. In addition, a simulated mock (SM) community dataset based on 73 bacterial genomes [12], comprising both MG and MT data was generated to serve as a means for ground truth-based assessment of IMP (details in section “Coupled metagenomic and metatranscriptomic datasets”). The SM dataset was devised given the absence of a standardized benchmarking dataset for coupled MG and MT data (this does solely exist for MG data as part of the CAMI initiative (http://www.cami-challenge.org)).

Analysis with IMP was carried out with the two available de novo assembler options for the co-assembly step (Fig. 1; Additional file 1: Figure S1), namely the default IDBA-UD assembler [22] (hereafter referred to as IMP) and the optional MEGAHIT assembler [23] (henceforth referred to as IMP-megahit). IMP was quantitatively assessed based on resource requirement and analytical capabilities. The analytical capabilities of IMP were evaluated based on data usage, output volume, and output quality. Accordingly, we assessed the advantages of the iterative assembly procedure as well as the overall data integration strategy.

#### Resource requirement and runtimes

IMP is an extensive pipeline that utilizes both MG and MT data within a reference-independent (assembly-based) analysis framework which renders it resource- and time-intensive. Therefore, we aimed to assess the required computational resource and runtimes of IMP.

All IMP-based runs on all datasets were performed on eight compute cores with 32 GB RAM per core and 1024 GB of total memory (section “Computational platforms”). IMP runtimes ranged from approximately 23 h (HF1) to 234 h (BG) and the IMP-megahit runtimes ranged from approximately 21 h (HF1) up to 281 h (BG). IMP was also executed on the Amazon cloud computing (AWS) infrastructure, using the HF1 dataset on a machine with 16 cores (section “Computational platforms”) whereby the run lasted approximately 13 h (refer to Additional file 1: Note S1 for more details). The analysis of IMP resulted in an increase in additional data of around 1.2–3.6 times the original input (Additional file 2: Table S1). Therefore, users should account for the disc space for both the final output and intermediate (temporary) files generated during an IMP run. Detailed runtimes and data generated for all the processed data sets are reported in Additional file 2: Table S1.

We further evaluated the effect of increasing resources using a small scale test dataset (section “Test dataset for runtime assessment”). The tests demonstrated that reduced runtimes are possible by allocating more threads to IMP-megahit (Additional file 2: Table S2). However, no apparent speed-up is achieved beyond allocation of eight threads, suggesting that this would be the optimal number of threads for this particular test dataset. Contrastingly, no speed-up was observed with additional memory allocation (Additional file 2: Table S3). Apart from the resources, runtime may also be affected by the input size, the underlying complexity of the dataset and/or behavior of individual tools within IMP.

#### Data usage: iterative assembly

De novo assemblies of MG data alone usually result in a large fraction of reads that are unmappable to the assembled contigs and therefore remain unused, thereby leading to suboptimal data usage [43, 58–60]. Previous studies have assembled sets of unmappable reads iteratively to successfully obtain additional contigs, leading to an overall increase in the number of predicted genes, which in turn results in improved data usage [43, 58–60]. Therefore, IMP uses an iterative assembly strategy to maximize NGS read usage. In order to evaluate the best iterative assembly approach for application within the IMP-based iterative co-assembly strategy, we attempted to determine the opportune number of assembly iterations in relation to assembly quality metrics and computational resources/runtimes.

The evaluation of the iterative assembly strategy was applied to MG and MT datasets. For both omic data types, it involved an “initial assembly” which is defined as the de novo assembly of all preprocessed reads. Additional iterations of assembly were then conducted using the reads that remained unmappable to the generated set of contigs (see section “Iterative single-omic assemblies” for details and parameters). The evaluation of the iterative assembly procedure was carried out based on the gain of additional contigs, cumulative contig length (bp), numbers of genes, and numbers of reads mappable to contigs. Table 1 shows the evaluation results of four representative data sets and Additional file 2: Table S4 shows the detailed results of the application of the approach to 11 datasets. In all the datasets evaluated, all iterations (1 to 3) after the initial assembly lead to an increase in total length of the assembly and numbers of mappable reads (Table 1; Additional file 2: Table S4). However, there was a notable decline in the number of additional contigs and predicted genes beyond the first iteration. Specifically, the first iteration of the MG assembly yielded up to 1.6% additional predicted genes while the equivalent on the MT data yielded up to 9% additional predicted genes (Additional file 2: Table S4). Considering the small increase (<1%) in the number of additional contigs and predicted genes beyond the first assembly iteration on one hand and the extended runtimes required to perform additional assembly iterations on the other hand, a generalized single iteration assembly approach was retained and implemented within the IMP-based iterative co-assembly (Fig. 1; Additional file 1: Figure S1). This approach aims to maximize data usage without drastically extending runtimes.

**Table 1:** Statistics of iterative assemblies performed on MG and MT datasets

		MG iterative assembly				MT iterative assembly			
Dataset	Iteration	Number of contigs (≥1 kb)	Cumulative length of assembled contigs (bp)	Number of predicted genes	Number of mapped reads	Number of contigs (all)	Cumulative length of assembled contigs (bp)	Number of predicted genes	Number of mapped reads
SM	Initial assembly	29063	182673343	186939	18977716	13436	8994518	13946	822718
	1	16	483336	329	9515	1286	502535	1272	16038
	2	6	213094	126	3425	48	18460	49	656
	3	1	86711	47	1536	0	0	0	0
HF1	Initial assembly	27028	145938650	154760	20715368	40989	45300233	66249	17525586
	1	15	966872	274	39839	2471	969614	2238	329400
	2	−1	26822	5	1276	26	10315	24	45642
	3	0	4855	0	172	3	1640	6	54788
WW1	Initial assembly	14815	77059275	81060	6513708	45118	22525759	49859	8423603
	1	28	3146390	1136	73511	2115	723904	1589	529441
	2	2	175634	114	4031	250	82048	201	13335
	3	1	30032	16	572	31	10280	18	65866
BG	Initial assembly	105282	545494441	593688	109949931	47628	27493690	60566	3754432
	1	417	10998269	3902	456821	3956	1397409	3061	130131
	2	5	335313	219	21647	717	250223	754	12766
	3	7	79022	20	2511	24	9060	22	5827

Results for all datasets available in Additional file 2: Table S2

Despite being developed specifically for the analysis of coupled MG and MT datasets, the iterative assembly can also be used for single omic datasets. To assess IMP’s performance on MG datasets, it was applied to the simulated MG datasets from the CAMI challenge (http://www.cami-challenge.org) and the results are shown in Additional file 1: Figure S2. IMP-based MG assembly using the MEGAHIT assembler on the CAMI dataset outperforms well-established MG pipelines such as MOCAT in all measures. In addition, IMP-based iterative assemblies also exhibit comparable performance to the gold standard assembly with regards to contigs ≥1 kb and number of predicted genes (http://www.cami-challenge.org). Detailed results of the CAMI assemblies are available in Additional file 2: Table S5. However, as no MT and/or coupled MG and MT datasets so far exist for the CAMI challenge, the full capabilities of IMP could not be assessed in relation to this initiative.

#### Data usage: multi-omic iterative co-assembly

In order to assess the advantages of integrated multi-omic co-assemblies of MG and MT data, IMP-based iterative co-assemblies (IMP and IMP-megahit) were compared against MG-only-based assemblies which include single-omic iterative MG assemblies generated using IMP (referred to as IMP_MG) and standard MG assemblies by MOCAT (hereafter referred to as MOCAT_MG) and MetAMOS (hereafter referred to as MetAMOS_MG). Furthermore, the available reads from the human fecal microbiome dataset (preprocessed with IMP) were mapped to the MetaHIT Integrated Gene Catalog (IGC) reference database [35] to compare the data usage of the different assembly procedures against a reference-dependent approach.

**Fig. 3:**
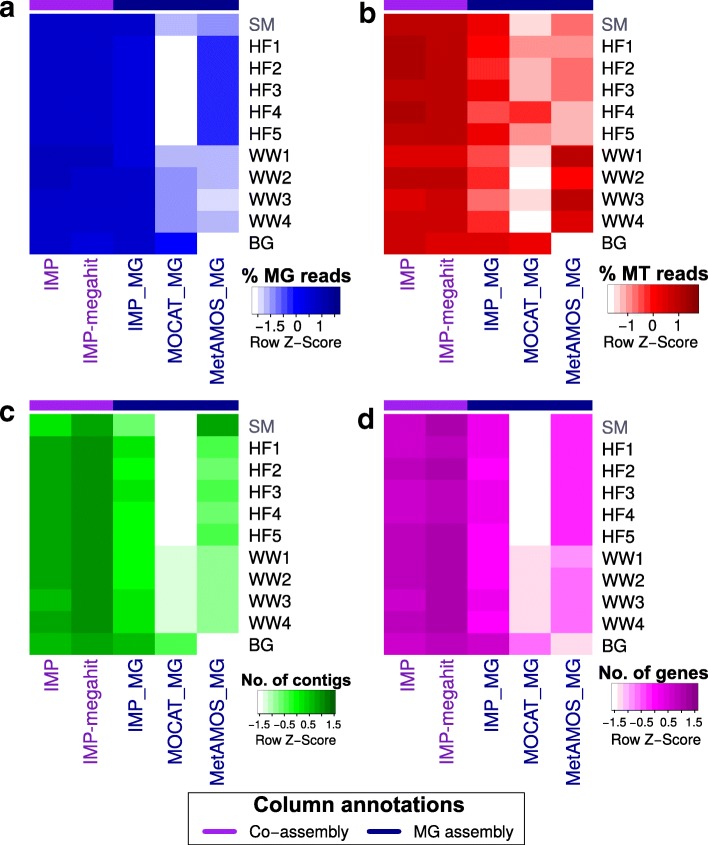
Assessment of data usage and output generated from co-assemblies compared to single-omic assemblies. Heat maps show (**a**) fractions of properly mapped MG read pairs, (**b**) fractions of properly mapped MT read pairs, (**c**) numbers of contigs ≥1 kb, and (**d**) numbers of unique predicted genes. IMP and IMP-megahit represent integrated multi-omic MG and MT iterative co-assemblies while IMP_MG, MOCAT_MG, and MetAMOS_MG represent single-omic MG assemblies. All numbers were row Z-score normalized for visualization. Detailed results available in Additional file 2: Table S5

IMP-based iterative co-assemblies consistently recruited larger fractions of properly paired MG (Fig. 3a) and/or MT (Fig. 3b) reads compared to single-omic assemblies. The resulting assemblies also produced larger numbers of contigs ≥1 kb (Fig. 3c), predicted non-redundant unique genes (Fig. 3d), and, even more important, complete genes as predicted with start and stop codon by Prodigal [61] (Additional file 2: Table S5). Using the reference genomes from the SM data as ground truth, IMP-based iterative co-assemblies resulted in up to 25.7% additional recovery of the reference genomes compared to the single-omic MG assemblies (Additional file 2: Table S5).

IMP-based iterative co-assemblies of the human fecal microbiome datasets (HF1–5) allowed recruitment of comparable fractions of properly paired MG reads and an overall larger fraction of properly paired MT reads compared to those mapping to the IGC reference database (Table 2). The total fraction (union) of MG or MT reads mapping to either IMP-based iterative co-assemblies and/or the IGC reference database was higher than 90%, thus demonstrating that the IMP-based iterative co-assemblies allow at least 10% of additional data to be mapped when using these assemblies in addition to the IGC reference database. In summary, the complementary use of de novo co-assembly of MG and MT datasets in combination with iterative assemblies enhances overall MG and MT data usage and thereby significantly increases the yield of useable information, especially when combined with comprehensive reference catalogs such as the IGC reference database.

**Table 2:** Mapping statistics for human microbiome samples

Reference	Average MG pairs mapping (%)	Average MT pairs mapping (%)
IGC	70.91	53.57
IMP	70.25	86.21
IMP-megahit	70.62	83.33
IMP_MG	68.08	58.54
MetAMOS_MG	57.31	37.34
MOCAT_MG	36.73	36.68
IMP + IGC	92.66	95.77
IMP-megahit + IGC	92.80	93.24

Average fractions (%) of properly paired reads from the human microbiome datasets (HF1–5) mapping to various references, including IMP-based iterative co-assemblies (IMP and IMP-megahit) and single-omic co-assemblies (IMP_MG, MetAMOS_MG, and MOCAT_MG) as well as the IGC reference database. IMP + IGC and IMP-megahit + IGC reports the total number of properly paired reads mapping to IMP-based iterative co-assemblies and/or the IGC reference database. Refer to Additional file 2: Table S3 for detailed information

#### Assembly quality: multi-omic iterative co-assembly

In order to compare the quality of the IMP-based iterative co-assembly procedure to simple co-assemblies, we compared the IMP-based iterative co-assemblies against co-assemblies generated using MetAMOS [10] (henceforth referred to as MetAMOS_MGMT) and MOCAT [34] (henceforth referred to as MOCAT_MGMT). Although MetAMOS and MOCAT were developed for MG data analysis, we extended their use for obtaining MG and MT co-assemblies by including both MG and MT read libraries as input (section “Execution of pipelines”). The assemblies were assessed based on contiguity (N50 length), data usage (MG and MT reads mapped), and output volume (number of contigs above 1 kb and number of genes; Additional file 2: Table S5). Only the SM dataset allowed for ground truth-based assessment by means of aligning the generated de novo assembly contigs to the original 73 bacterial genomes used to simulate the data set (section “Simulated coupled metagenomic and metatranscriptomic dataset”) [12, 54]. This allowed the comparison of two additional quality metrics, i.e., the recovered genome fraction and the composite performance metric (CPM) proposed by Deng et al. [62].

**Fig. 4:**
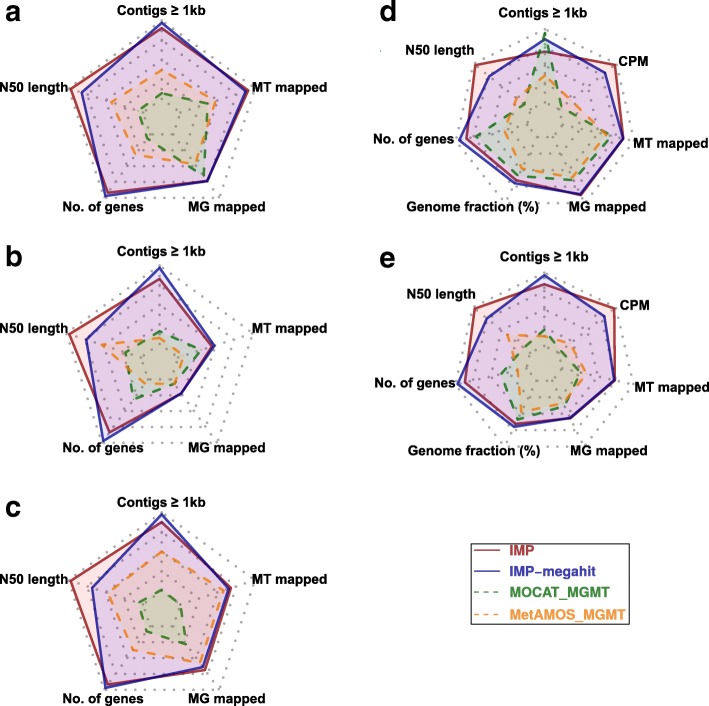
Assessment of the IMP-based iterative co-assemblies in comparison to MOCAT- and MetAMOS-based co-assemblies. Radar charts summarizing the characteristics of the co-assemblies generated using IMP, MetAMOS, and MOCAT pipelines on: **a** human fecal microbiome, **b** wastewater sludge community, **c** biogas reactor, **d** simulated mock community. IMP co-assemblies were performed with two de novo assembler options, IDBA_UD and MEGAHIT, whereas MetAMOS and MOCAT were executed using default settings. Assessment metrics within the radar charts include number of contigs ≥1 kb, N50 length (contiguity, cutoff 500 bp), number of predicted genes (unique), and fraction of properly mapped MG and MT read pairs. N50 statistics are reported using a 500-bp cutoff. Additional ground truth assessments for simulated mock dataset included recovered genome fractions (%) and the composite performance metric (CPM) score with a cutoff of 500 bp [62]. **e** Summary radar chart reflecting the cumulative measures and mean fraction of properly mapped MG and MT read pairs from all analyzed 11 datasets while incorporating ground truth-based measures from the simulated mock dataset. Higher values within the radar charts (furthest from center) represent better performance. Detailed information on the assembly assessments is available in Additional file 2: Table S5

Assessments based on real datasets demonstrate comparable performance between IMP and IMP-megahit while both outperform MetAMOS_MGMT and MOCAT_MGMT in all measures (Fig. 4a–c). The ground truth assessment using the SM dataset shows that IMP-based iterative co-assemblies are effective in recovering the largest fraction of the original reference genomes while achieving a higher CPM score compared to co-assemblies from the other pipelines. Misassembled (chimeric) contigs are a legitimate concern within extensive de novo assembly procedures such as the IMP-based iterative co-assembly. It has been previously demonstrated that highly contiguous assemblies (represented by high N50 lengths) tend to contain higher absolute numbers of misassembled contigs compared to highly fragmented assemblies, thereby misrepresenting the actual quality of assemblies [38, 62, 63]. Therefore, the CPM score was devised as it represents a normalized measure reflecting both contiguity and accuracy for a given assembly [62]. Based on the CPM score, both IMP and IMP-megahit yield assemblies that balance high contiguity with accuracy and thereby outperform the other methods (Fig. 4c, d). In summary, cumulative measures of numbers of contigs ≥1 kb, N50 lengths, numbers of unique genes, recovered genome fractions (%), and CPM scores (the latter two were only calculated for the SM dataset), as well as the mean fractions (%) of mappable MG and MT reads, show that the IMP-based iterative co-assemblies (IMP and IMP-megahit) clearly outperform all other available methods (Fig. 4e; Additional file 2: Table S5).

### Use-cases of integrated metagenomic and metatranscriptomic analyses in IMP

The integration of MG and MT data provides unique opportunities for uncovering community- or population-specific traits, which cannot be resolved from MG or MT data alone. Here we provide two examples of insights gained through the direct inspection of results provided by IMP.

#### Tailored preprocessing and filtering of MG and MT data

The preprocessing of the datasets HF1–5 included filtering of human-derived sequences, while the same step was not necessary for the non-human-derived datasets, WW1–4 and BG. MT data analyzed within this article included RNA extracts which were not subjected to wet-lab rRNA depletion, i.e., BG [29], and samples which were treated with wet-lab rRNA removal kits (namely HF1–5 [28] and WW1–4 [43]). Overall, the removal of rRNA pairs from the MT data showed a large variation, ranging from as low as 0.51% (HF5) to 60.91% (BG), demonstrating that wet-lab methods vary in terms of effectiveness and highlighting the need for such MT-specific filtering procedures (Additional file 1: Note S2; Additional file 2: Table S6).

#### Identification of RNA viruses

To identify differences in the information content of MG and MT complements, the contigs generated using IMP were inspected with respect to coverage by MG and MT reads (Additional file 2: Table S7). In two exemplary datasets HF1 and WW1, a small fraction of the contigs resulted exclusively from MT data (Additional file 2: Table S7). Longer contigs (≥1 kb) composed exclusively of MT reads and annotated with known viral/bacteriophage genes were retained for further inspection (Table 3; complete list contigs in Additional file 2: Table S8 and S9). A subsequent sequence similarity search against the NCBI NR nucleotide database [64] of these candidate contigs revealed that the longer contigs represent almost complete genomes of RNA viruses (Additional file 2: Table S10 and S11). This demonstrates that the incorporation of MT data and their contrasting to the MG data allow the identification and recovery of nearly complete RNA viral genomes, thereby allowing their detailed future study in a range of microbial ecosystems.

**Table 3:** Contigs with a likely viral/bacteriophage origin/function reconstructed from the metatranscriptomic data

Sample	Contig ID*	Contig length	Average contig depth of coverage	Gene product	Average gene depth of coverage
HF1	Contig_34	6468	20927	Virus coat protein (TMV like)	30668
				Viral movement protein (MP)	26043
				RNA-dependent RNA polymerase	22578
				Viral methyltransferase	18817
	Contig_13948	2074	46	RNA-dependent RNA polymerase	41
				Viral movement protein (MP)	56
WW2	Contig_6405	4062	46	Tombusvirus p33	43
				Viral RNA-dependent RNA polymerase	42
				Viral coat protein (S domain)	36
	Contig_7409	3217	21	Viral RNA-dependent RNA polymerase	18
				Viral coat protein (S domain)	21
	Contig_7872	2955	77	Hypothetical protein	112
				Phage maturation protein	103

*Contigs of ≥1 kb and average depth of coverage ≥20 were selected

#### Identification of populations with apparent high transcriptional activity

To further demonstrate the unique analytical capabilities of IMP, we aimed to identify microbial populations with a high transcriptional activity in the HF1 human fecal microbiome sample. Average depth of coverage at the contig- and gene-level is a common measure used to evaluate the abundance of microbial populations within communities [14, 16, 43]. The IMP-based integrative analysis of MG and MT data further extends this measure by calculation of average MT to MG depth of coverage ratios, which provide information on transcriptional activity and which can be visualized using augmented VizBin maps [56].

**Fig. 5:**
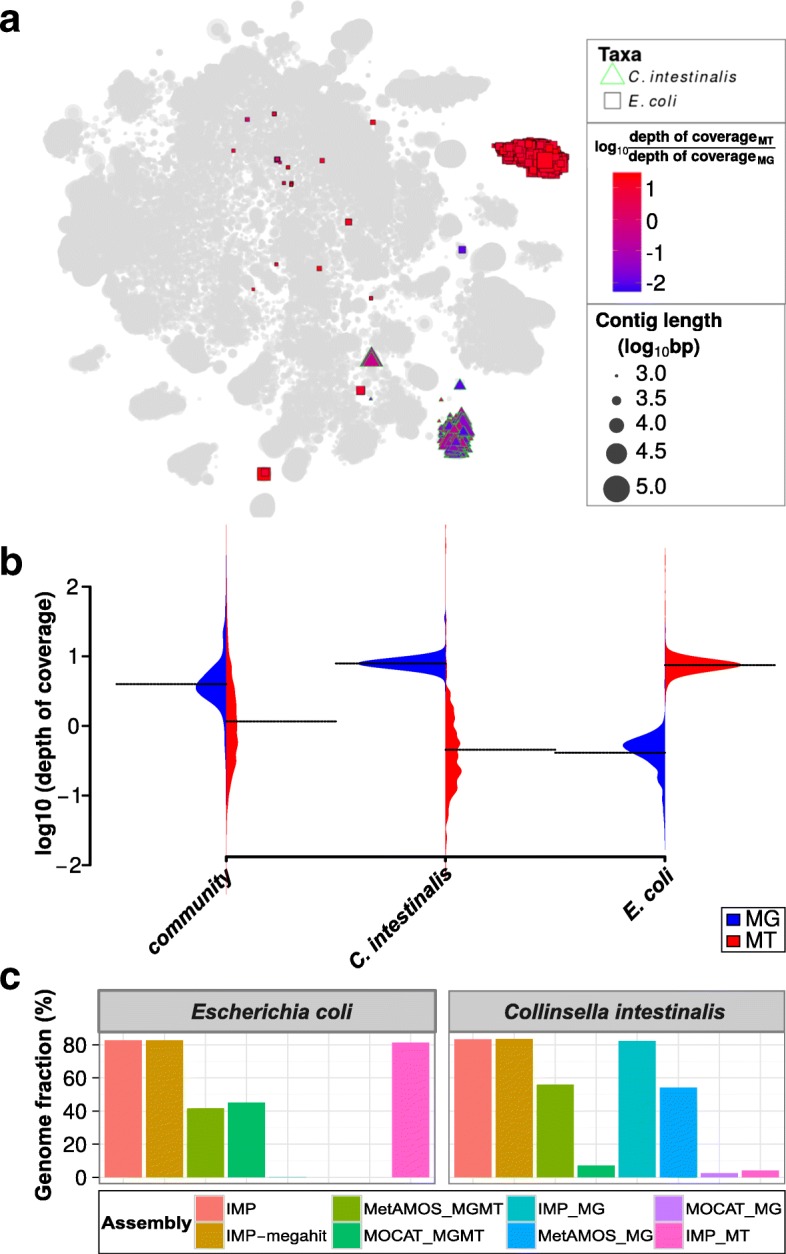
Metagenomic and metatranscriptomic data integration of a human fecal microbiome. **a** Augmented VizBin map highlighting contig subsets with sequences that are most similar to *Escherichia coli* P12b and *Collinsella intestinalis* DSM 13280 genomes. **b** Beanplots representing the densities of metagenomic (*MG*) and metatranscriptomic (*MT*) average contig-level depth of coverage for the entire microbial community and two subsets (population-level genomes) of interest. The *dotted lines* represent the mean. **c** Recovered portion of genomes of the aforementioned taxa based on different single-omic assemblies and multi-omic co-assemblies (Additional file 2: Table S5)

In our example, one particular cluster of contigs within the augmented VizBin maps exhibited high MT to MG depth of coverage ratios (Additional file 1: Figure S3). The subset of contigs within this cluster aligned to the genome of the *Escherichia coli* P12B strain (henceforth referred to as *E. coli*). For comparison, we also identified a subset, which was highly abundant at the MG level (lower MT to MG ratio), which aligned to the genome of *Collinsella intestinalis* DSM 13280 strain (henceforth referred to as *C. intestinalis*). Based on these observations, we highlighted the subsets of these contigs in an augmented VizBin map (Fig. 5a). The *C. intestinalis* and *E. coli* subsets are mainly represented by clear peripheral clusters which exhibit consistent intra-cluster MT to MG depth of coverage ratios (Fig. 5a). The subsets were manually inspected in terms of their distribution of average MG and MT depths of coverage and were compared against the corresponding distributions for all contigs. The MG-based average depths of coverage of the contigs from the entire community exhibited a bell-shape like distribution, with a clear peak (Fig. 5b). In contrast, MT depths of coverage exhibited more spread, with a relatively low mean (compared to MG distribution) and no clear peak (Fig. 5b). The *C. intestinalis* subset displays similar distributions to that of the entire community, whereas the *E. coli* subset clearly exhibits unusually high MT-based and low MG-based depths of coverage (Fig. 5b). Further inspection of the individual omic datasets revealed that the *E. coli* subset was not covered by the MG contigs, while approximately 80% of the *E. coli* genome was recoverable from a single-omic MT assembly (Fig. 5c). In contrast, the *C. intestinalis* subset demonstrated genomic recovery in all co-assemblies (IMP, IMP-megahit, MOCAT_MGMT, MetAMOS_MGMT) and the single-omic MG assemblies (IMP_MG, MOCAT_MG, MetAMOS_MG; Fig. 5c).

As noted by the authors of the original study by Franzosa et al. [28], the cDNA conversion protocol used to produce the MT data is known to introduce approximately 1–2% of *E. coli* genomic DNA into the cDNA as contamination which is then reflected in the MT data. According to our analyses, 0.12% of MG reads and 1.95% of MT reads derived from this sample could be mapped onto the *E. coli* contigs, which is consistent with the numbers quoted by Franzosa et al. [28].

Consistent recovery of the *E. coli* genome was also observed across all other assemblies of the human fecal microbiome datasets (HF2–5) which included their respective MT data (Additional file 1: Figure S4; Additional file 2: Table S12). The integrative analyses of MG and MT data within IMP enables users to efficiently highlight notable cases such as this and to further investigate inconsistencies and/or interesting characteristics within these multi-omic datasets.

## Discussion

The microbiome analysis workflow of IMP is unique in that it allows the integrated analysis of MG and MT data. To the best of our knowledge, IMP represents the only pipeline that spans the preprocessing of NGS reads to the binning of the assembled contigs, in addition to being the first automated pipeline for reproducible reference-independent metagenomic and metatranscriptomic data analysis. Although existing pipelines such as MetAMOS or MOCAT may be applied to perform co-assemblies of MG and MT data [44], these tools do not include specific steps for the two data types in their pre- and post-assembly procedures, which is important given the disparate nature of these datasets. The use of Docker promotes reproducibility and sharing, thereby allowing researchers to precisely replicate the IMP workflow with relative ease and with minimal impact on overall performance of the employed bioinformatic tools [29, 46–48]. Furthermore, static websites will be created and associated with every new version of IMP (Docker image), such that users will be able to download and launch specific versions of the pipeline to reproduce the work of others. Thereby, IMP enables standardized comparative studies between datasets from different labs, studies, and environments. The open source nature of IMP encourages a community-driven effort to contribute to and further improve the pipeline. Snakemake allows the seamless integration of Python code and shell (bash) commands and the use of *make* scripting style, which are arguably some of the most widely used bioinformatic scripting languages. Snakemake also supports parallel processing and the ability to interoperate with various tools and/or web services [49, 51]. Thus, users will be able to customize and enhance the features of the IMP according to their analysis requirements with minimal training/learning.

Quality control of NGS data prior to de novo assemblies has been shown to increase the quality of downstream assembly and analyses (predicted genes) [63]. In addition to standard preprocessing procedures (i.e., removal of low quality reads, trimming of adapter sequences and removal), IMP incorporates additional tailored and customizable filtering procedures which account for the different sample and/or omic data types. For instance, the removal of host-derived sequences in the context of human microbiomes is required for protecting the privacy of study subjects. The MT-specific in silico rRNA removal procedure yielded varying fractions of rRNA reads between the different MT datasets despite the previous depletion of rRNA (section “Tailored preprocessing and filtering of MG and MT data”), indicating that improvements in wet-lab protocols are necessary. Given that rRNA sequences are known to be highly similar, they are removed in IMP in order to mitigate any possible misassemblies resulting from such reads and/or regions [65, 66]. In summary, IMP is designed to perform stringent and standardized preprocessing of MG and MT data in a data-specific way, thereby enabling efficient data usage and resulting in high-quality output.

It is common practice that MG and MT reads are mapped against a reference (e.g., genes, genomes, and/or MG assemblies) [28, 29, 40] prior to subsequent data interpretation. However, these standard practices lead to suboptimal usage of the original data. IMP enhances overall data usage through its specifically tailored iterative co-assembly procedure, which involves four measures to achieve better data usage and yield overall larger volumes of output (i.e., a larger number of contigs ≥1 kb and predicted unique and complete genes).

First, the iterative assembly procedure leads to increases in data usage and output volume in each additional iterative assembly step (section “Data usage: iterative assembly”). The exclusion of mappable reads in each iteration of the assembly serves as a means of partitioning the data, thereby reducing the complexity of the data and overall, resulting in a higher cumulative volume of output [60, 63, 67].

Second, the initial assembly of MT-based contigs enhances the overall assembly, as transcribed regions are covered much more deeply and evenly in MT data, resulting in better assemblies for these regions [43]. The MT-based contigs represent high-quality scaffolds for the subsequent co-assembly with MG data.

Third, the co-assembly of MG and MT data allows the integration of these two data types while resulting in a larger number of contigs and predicted complete genes against which, in turn, a substantially higher fraction of reads can be mapped (section “Data usage: multi-omic iterative co-assembly”). Furthermore, the analyses of the human fecal microbiome datasets (HF1–5) demonstrate that the numbers of MG reads mapping to the IMP-based iterative co-assemblies for each sample are comparable to the numbers of reads mapping to the comprehensive IGC reference database (Table 2). Previously, only fractions of 74–81% of metagenomic reads mapping to the IGC have been reported [35]. However, such numbers have yet to be reported for MT data, in which case we observe lower mapping rates to the IGC reference database (35.5–70.5%) compared to IMP-based assemblies (Additional file 2: Table S3). This may be attributed to the fact that the IGC reference database was generated from MG-based assemblies only, thus creating a bias [35]. Moreover, an excess of 90% of MG and MT reads from the human fecal datasets (HF1–5) are mappable to either the IGC reference database and/or IMP-based iterative co-assemblies, emphasizing that a combined reference-based and IMP-based integrated-omics approach vastly improves data usage (Table 2). Although large fractions of MG and/or MT reads can be mapped to the IGC, a significant advantage of using a de novo reference-independent approach lies within the fact that reads can be linked to genes within their respective genomic context and microbial populations of origin. Exploiting the maximal amount of information is especially relevant for microbial communities with small sample sizes and which lack comprehensive references such as the IGC reference database.

Fourth, the assembly refinement step via a contig-level assembly with cap3 improves the quality of the assemblies by reducing redundancy and increasing contiguity by collapsing and merging contigs (section “Assembly quality: multi-omic iterative co-assembly”). Consequently, our results support the described notion that the sequential use of multi-*k*mer-based de Bruijn graph assemblers, such as IDBA-UD and MEGAHIT, with overlap-layout-consensus assemblers, such as cap3, result in improved MG assemblies [38, 62] but importantly also extend this to MG and MT co-assemblies.

When compared to commonly used assembly strategies, the IMP-based iterative co-assemblies consisted of a larger output volume while maintaining a relatively high quality of the generated contigs. High-quality assemblies yield higher quality taxonomic information and gene annotations while longer contigs (≥1 kb) are a prerequisite for unsupervised population-level genome reconstruction [14, 19, 56] and subsequent multi-omics data integration [39, 43, 44]. Throughout all the different comparative analyses which we performed, IMP performed more consistently across all the different datasets when compared to existing methods, thereby emphasizing the overall stability and broad range of applicability of the method (section “Assembly quality: multi-omic iterative co-assembly”).

Integrated analyses of MG and MT data with IMP provide the opportunity for analyses that are not possible based on MG data alone, such as the detection of RNA viruses (section “Identification of RNA viruses”) and the identification of transcriptionally active populations (section “Identification of populations with apparent high transcriptional activity”). The predicted/annotated genes may be used for further analyses and integration of additional omic datasets, most notably metaproteomic data [39, 43, 44]. Furthermore, the higher number of complete genes improves the downstream functional analysis, because the read counts per gene will be much more accurate when having full length transcript sequences and will increase the probability to identify peptides. More specifically, the large number of predicted genes may enhance the usage of generated metaproteomic data, allowing more peptides, and thus proteins, to be identified.

## Conclusions

IMP represents the first self-contained and standardized pipeline developed to leverage the advantages associated with integrating MG and MT data for large-scale analyses of microbial community structure and function in situ [4, 6]. IMP performs all the necessary large-scale bioinformatic analyses, including preprocessing, assembly, binning (automated), and analyses within an automated, reproducible, and user-friendly pipeline. In addition, we demonstrate that IMP vastly enhances data usage to produce high-volume and high-quality output. Finally, the combination of open development and reproducibility should promote the general paradigm of reproducible research within the microbiome research community.

## Methods

The details of the IMP workflow, implementation, and customizability are described in further detail. We also describe the additional analyses carried out for assessment and benchmarking of IMP.

### Details of the IMP implementation and workflow

A Python (v3) wrapper script was implemented for user-friendly execution of IMP via the command line. The full list of dependencies, parameters (see below), and documentation is available on the IMP website (http://r3lab.uni.lu/web/imp/doc.html). Although IMP was designed specifically for integrated analysis of MG and MT data, it can also be used for single MG or MT analyses as an additional functionality.

#### Reproducibility

IMP is implemented around a Docker container that runs the Ubuntu 14.04 operating system, with all relevant dependencies. Five mounting points are defined for the Docker container with the -v option: i) input directory, ii) output directory, iii) database directory, iv) code directory, and v) configuration file directory. Environment variables are defined using the -e parameter, including: i) paired MG data, ii) paired MT data, and iii) configuration file. The latest IMP Docker image will be downloaded and installed automatically upon launching the command, but users may also launch specific versions based on tags or use modified/customized versions of their local code base (documentation at http://r3lab.uni.lu/web/imp/doc.html).

#### Automation and modularity

Automation of the workflow is achieved using Snakemake 3.4.2 [49, 51], a Python-based make language implemented specifically for building reproducible bioinformatic workflows and pipelines. Snakemake is inherently modular and thus allows various features to be implemented within IMP, including the options of i) executing specific/selected steps within the pipeline, ii) check-pointing, i.e., resuming analysis from a point of possible interruption/termination, iii) analysis of single-omic datasets (MG or MT). For more details regarding the functionalities of IMP, please refer to the documentation of IMP (http://r3lab.uni.lu/web/imp/doc.html).

#### Input data

The input to IMP includes MG and/or MT FASTQ paired files, i.e., pairs-1 and pairs-2 are in individual files. The required arguments for the IMP wrapper script are metagenomic paired-end reads (“-m” options) and/or metatranscriptomic paired-end reads (“-t” option) with the specified output folder (“-o” option). Users may customize the command with the options and flags described in the documentation (http://r3lab.uni.lu/web/imp/doc.html) and in the “Customization and further development” section.

#### Trimming and quality filtering

Trimmomatic 0.32 [52] is used to perform trimming and quality filtering of MG and MT Illumina paired-end reads, using the following parameters: ILLUMINACLIP:TruSeq3-PE.fa:2:30:10; LEADING:20; TRAILING:20; SLIDINGWINDOW:1:3; MAXINFO:40:0.5; MINLEN:40. The parameters may be tuned via the command line or within the IMP config file. The output from this step includes retained paired-end and single-end reads (mate discarded), which are all used for downstream processes. These parameters are configurable in the IMP config file (section “Customization and further development”)

#### Ribosomal RNA filtering

SortMeRNA 2.0 [68] is used for filtering rRNA from the MT data. The process is applied on FASTQ files for both paired- and single-end reads generated from the trimming and quality filtering step. Paired-end FASTQ files are interleaved prior to running SortMeRNA. If one of the mates within the paired-end read is classified as an rRNA sequence, then the entire pair is filtered out. After running SortMeRNA, the interleaved paired-end output is split into two separate paired-end FASTQ files. The filtered sequences (without rRNA reads) are used for the downstream processes. All available databases provided within SortMeRNA are used for filtering and the maximum memory usage parameter is set to 4 GB (option: “-m 4000”), which can be adjusted in the IMP config file (section “Customization and further development”).

#### Read mapping

The read mapping procedure is performed using the bwa mem aligner [69] with settings: “ -v 1” (verbose output level), “-M” (Picard compatibility) introducing an automated samtools header using the “-R” option [69]. Paired- and single-end reads are mapped separately and the resulting alignments are merged (using samtools merge [70]). The output is written as a binary aligment map (BAM) file. Read mapping is performed at various steps in the workflow, including: i) screening for host or contaminant sequences (section “Screening host or contaminant sequences”), ii) recruitment of unmapped reads within the IMP-based iterative co-assembly (section “Extracting unmapped reads”), and iii) mapping of preprocessed MG and MT reads to the final contigs. The memory usage is configurable in the IMP config file (section “Customization and further development”).

#### Extracting unmapped reads

The extraction of unmapped reads (paired- and single-end) begins by mapping reads to a given reference sequence (section “Read mapping”). The resulting BAM file is used as input for the extraction of unmapped reads. A set of paired-end reads are considered unmappable if both or either one of the mates do not map to the given reference. The unmapped reads are converted from BAM to FASTQ format using samtools [70] and BEDtools 2.17.0—bamToFastq utility [71]. Similarly, unmapped single-end reads are also extracted from the alignment information.

#### Screening host or contaminant sequences

By default, the host/contaminant sequence screening is performed by mapping both paired- and single-end reads (section “Read mapping”) onto the human genome version 38 (http://www.ncbi.nlm.nih.gov/projects/genome/assembly/grc/), followed by extraction of unmapped reads (section “Extracting unmapped reads”). Within the IMP command line, users are provided with the option of i) excluding this procedure with the “- -no-filtering” flag, ii) using other sequence(s) for screening by providing the FASTA file (or URL) using “- -screen” option, or iii) specifying it in the configuration file (section “Customization and further development”).

#### Parameters of the IMP-based iterative co-assembly

The IMP-based iterative co-assembly implements MEGAHIT 1.0.3 [23] as the MT assembler while IDBA-UD 1.1.1 [22] is used as the default co-assembler (MG and MT), with MEGAHIT [23] as an alternative option for the co-assembler (specified by the “-a” option of the IMP command line). All de novo assemblies are performed on *k*mers ranging from 25-mers to 99-mers, with an incremental step of four. Accordingly, the command line parameters for IDBA-UD are “- -mink 25 - -maxk 99 - -step 4 - -similar 0.98 - -pre-correction” [22]. Similarly, the command line parameters for MEGAHIT are “- -k-min 25 - -k-max 99 - -k-step 4”, except for the MT assemblies which are performed with an additional “- -no-bubble” option to prevent merging of bubbles within the assembly graph [23]. Furthermore, contigs generated from the MT assembly are used as “long read” input within the “-l” flag of IDBA-UD or “-r” flag of MEGAHIT [22, 23]. *K*mer ranges for the IDBA-UD and MEGAHIT can be adjusted/specified in the configuration file (section “Customization and further development”). Cap3 is used to reduce the redundancy and improve contiguity of the assemblies using a minimum alignment identity of 98% (“-p 0.98”) with a minimum overlap of 100 bases (“-o 100”), which are adjustable in the configuration file (section “Customization and further development”). Finally, the extraction of reads that are unmappable to the initial MT assembly and initial co-assembly is described in the “Extracting unmapped reads” section.

#### Annotation and assembly quality assessment

Prokka 1.11 [55] with the “- -metagenome” setting is used to perform functional annotation. The default BLAST and HMM databases of Prokka are used for the functional annotation. Custom databases may be provided by the user (refer to the “Databases” and “Customization and further development” sections for details).

MetaQUAST 3.1 [54] is used to perform taxonomic annotation of contigs with the maximum number of downloadable reference genomes set to 20 (“- -max-ref-number 20”). In addition, MetaQUAST provides various assembly statistics. The maximum number of downloadable reference genomes can be changed in the IMP config file (see “Customization and further development” for details).

#### Depth of coverage

Contig- and gene-wise depth of coverage values are calculated (per base) using BEDtools 2.17.0 [71] and aggregated (by average) using awk, adapted from the CONCOCT code [16] (script: map-bowtie2-markduplicates.sh; https://github.com/BinPro/CONCOCT) and is non-configurable.

#### Variant calling

The variant calling procedure is performed using Samtools 0.1.19 [70] (mpileup tool) and Platypus 0.8.1 [72], each using their respective default settings and which are non-configurable. The input is the merged paired- and single-end read alignment (BAM) against the final assembly FASTA file (section “Read mapping”). The output files from both the methods are indexed using tabix and compressed using gzip. No filtering is applied to the variant calls, so that users may access all the information and filter it according to their requirements. The output from samtools mpileup is used for the augmented VizBin visualization.

#### Non-linear dimensionality reduction of genomic signatures

VizBin [56] performs non-linear dimensionality reduction of genomic signatures onto contigs ≥1 kb, using default settings, to obtain two-dimensional embeddings. Parameters can be modified in the IMP config file (section “Customization and further development”).

#### Automated binning

Automated binning of the assembled contigs is performed using MaxBin 2.0. Default setting are applied and paired-end reads are provided as input for abundance estimation [20]. The sequence length cutoff is set to be same as VizBin (section “Non-linear dimensionality reduction of genomic signatures”) and is customizable using the config file (section “Customization and further development”).

#### Visualization and reporting

IMP compiles the multiple summaries and visualizations into a HTML report [57]. FASTQC [73] is used to visualize the quality and quantity of reads before and after preprocessing. MetaQUAST [54] is used to report assembly quality and taxonomic associations of contigs. A custom script is used to generate KEGG-based [74] functional Krona plots by running KronaTools [75] (script: genes.to.kronaTable.py, GitHub URL: https://github.com/EnvGen/metagenomics-workshop). Additionally, VizBin output (two-dimensional embeddings) is integrated with the information derived from the IMP analyses, using a custom R script for analysis and visualization of the augmented maps. The R workspace image is saved such that users are able to access it for further analyses. All the steps executed within an IMP run, including parameters and runtimes, are summarized in the form of a workflow diagram and a log-file. The visualization script is not configurable.

#### Output

The output generated by IMP includes a multitude of large files. Paired- and single-end FASTQ files of preprocessed MG and MT reads are provided such that the user may employ them for additional downstream analyses. The output of the IMP-based iterative co-assembly consists of a FASTA file, while the alignments/mapping of MG and MT preprocessed reads to the final co-assembly are also provided as BAM files, such that users may use these for further processing. Predicted genes and their respective annotations are provided in the various formats produced by Prokka [55]. Assembly quality statistics and taxonomic annotations of contigs are provided as per the output of MetaQUAST [54]. Two-dimensional embeddings from the NLDR-GS are provided such that they can be exported to and further curated using VizBin [56]. Additionally, abundance and expression information is represented by contig- and gene-level average depth of coverage values. MG and MT genomic variant information (VCF format), including both SNPs and INDELs (insertions and deletions), is also provided. The results of the automated binning using MaxBin 2.0 [20] are provided in a folder which contains the default output from the program (i.e., fasta files of bins and summary files).

The HTML reports [57], e.g., HTML S1 and S2, compile various summaries and visualizations, including, i) augmented VizBin maps, ii) MG- and MT-level functional Krona charts [75], iii) detailed schematics of the steps carried out within the IMP run, iv) list of parameters and commands, and v) additional reports (FASTQC report [73], MetaQUAST report [54]). Please refer to the documentation of IMP for a detailed list and description of the output (http://r3lab.uni.lu/web/imp/doc.html).

#### Databases

The IMP database folder (db) contains required databases required for IMP analysis. The folder contains the following subfolders and files with their specific content:

i. adapters folder — sequencing adapter sequences. Default version contains all sequences provided by Trimmomatic version 0.32 [52]
ii. cm, genus, hmm, and kingdom folders — contains databases provided by Prokka 1.11 [55]. Additional databases may be added into the corresponding folders as per the instructions in the Prokka documentation (https://github.com/tseemann/prokka#databases)
iii. sortmerna folder — contains all the databases provided in SortMeRNA 2.0 [68]. Additional databases may be added into the corresponding folders as per the instructions in the SortMeRNA documentation (http://bioinfo.lifl.fr/RNA/sortmerna/code/SortMeRNA-user-manual-v2.0.pdf)
iv. ec2pathways.txt — enzyme commission (EC) number mapping of amino acid sequences to pathways
v. pathways2hierarchy.txt — pathway hierarchies used to generated for KEGG-based functional Krona plot (section “Visualization and reporting”)

#### Customization and further development

Additional advanced parameters can be specified via the IMP command line, including specifying a custom configuration file (“-c” option) and/or specifying a custom database folders (“-d” option). Threads (“- -threads”) and memory allocation (“- -memcore” and “- -memtotal”) can be adjusted via the command line and the configuration file. The IMP launcher script provides a flag (“- -enter”) to launch the Docker container interactively and the option to specify the path to the customized source code folder (“-s” option). These commands are provided for development and testing purposes (described on the IMP website and documentation: http://r3lab.uni.lu/web/imp/doc.html). Further customization is possible using a custom configuration file (JSON format). The customizable options within the JSON file are specified in individual subsections within the “Details of the IMP implementation and workflow” section. Finally, the open source implementation of IMP allows users to customize the Docker image and source code of IMP according to their requirements.

### Iterative single-omic assemblies

In order to determine the opportune number of iterations within the IMP-based iterative co-assembly strategy an initial assembly was performed using IMP preprocessed MG reads with IDBA-UD [22]. Cap3 [53] was used to further collapse the contigs and reduce the redundancy of the assembly. This initial assembly was followed by a total of three assembly iterations, whereby each iteration was made up of four separate steps: i) extraction of reads unmappable to the previous assembly (using the procedure described in the “Extracting unmapped reads” section), ii) assembly of unmapped reads using IDBA-UD [22], iii) merging/collapsing the contigs from the previous assembly using cap3 [53], and iv) evaluation of the merged assembly using MetaQUAST [54]. The assembly was evaluated in terms of the per-iteration increase in mappable reads, assembly length, numbers of contigs ≥1 kb, and numbers of unique genes.

Similar iterative assemblies were also performed for MT data using MEGAHIT [23], except CD-HIT-EST [76] was used to collapse the contigs at ≥95% identity (“-c 0.95”) while MetaGeneMark [77] was used to predict genes. The parameters and settings of the other programs were the same as those defined in the “Details of the IMP implementation and workflow” section.

The aforementioned procedures were applied to all the datasets analyzed within this article. The merged contig sets (non-redundant) from the first iteration of both the MG and MT iterative assemblies were selected to represent the IMP single-omics assemblies (IMP_MG and IMP_MT) and were compared against co-assemblies.

### Execution of pipelines

MetAMOS v1.5rc3 was executed using default settings. MG data were provided as input for single-omic assemblies (MetAMOS_MG) while MG and MT data were provided as input for multi-omic co-assemblies (MetAMOS_MGMT). All computations using MetAMOS were set to use eight computing cores (“-p 8”).

MOCAT v1.3 (MOCAT.pl) was executed using default settings. Paired-end MG data were provided as input for single-omic assemblies (MOCAT_MG) while paired-end MG and MT data were provided as input for multi-omic co-assemblies (MOCAT_MGMT). All computations using MOCAT were set to use eight computing cores (“-cpus 8”). Paired-end reads were first preprocessed using the read_trim_filter step of MOCAT (“-rtf”). For the human fecal microbiome datasets (HF1–5), the preprocessed paired- and single-end reads were additionally screened for human genome-derived sequences (“-s hg19”). The resulting reads were afterwards assembled with default parameters (“-gp assembly -r hg19”) using SOAPdenovo.

IMP v1.4 was executed for each dataset using different assemblers for the co-assembly step: i) default setting using IDBA-UD, and ii) MEGAHIT (“-a megahit”). Additionally, the analysis of human fecal microbiome datasets (HF1–5) included the preprocessing step of filtering human genome sequences, which was omitted for the wastewater sludge datasets (WW1–4) and the biogas (BG) reactor dataset. Illumina TruSeq2 adapter trimming was used for wastewater dataset preprocessing since the information was available. Computation was performed using eight computing cores (“- -threads 8”), 32 GB memory per core (“- -memcore 32”) and total memory of 256 GB (“- -memtotal 256 GB”). The customized parameters were specified in the IMP configuration file (exact configurations listed in the HTML reports [57]). The analysis of the CAMI datasets were carried using the MEGAHIT assembler option (“-a megahit”), while the other options remained as default settings.

In addition, IMP was also used on a small scale dataset to evaluate performance of increasing the number of threads from 1 to 32 and recording the runtime (“time” command). IMP was launched on the AWS cloud computing platform running the MEGAHIT as the assembler (“-a megahit”) with 16 threads (“- -threads 16”) and 122 GB of memory (“- -memtotal 122”).

### Data usage assessment

Preprocessed paired-end and single-end MG and MT reads from IMP were mapped (section Read mapping) onto the IMP-based iterative co-assemblies and IMP_MG assembly. Similarly, preprocessed paired-end and single-end MG and MT reads from MOCAT were mapped onto the MOCAT co-assembly (MOCAT_MGMT) and the MOCAT single-omic MG assembly (MOCAT_MG). MetAMOS does not retain single-end reads; therefore, preprocessed MG and MT paired-end reads from MetAMOS were mapped onto the MetAMOS co-assembly (MetAMOS_MGMT) and MetAMOS single-omic MG assembly (MetAMOS_MG).

Preprocessed MG and MT reads from the human fecal datasets (HF1–5) were mapped using the same parameters described in the “Read mapping” section to the IGC reference database [35] for evaluation of a reference-based approach. Alignment files of MG and MT reads mapping to the IMP-based iterative co-assemblies and the aforementioned alignments to the IGC reference database were used to report the fractions of properly paired reads mapping in either IMP-based iterative co-assembly, IGC reference database, or both. These fractions were then averaged across all the human fecal datasets (HF1–5).

### Assembly assessment and comparison

Assemblies were assessed and compared using MetaQUAST by providing contigs (FASTA format) from all different (single- and multi-omic) assemblies of the same dataset as input [54]. The gene calling function (“-f”) was utilized to obtain the number of genes which were predicted from the various assemblies. An additional parameter within MetaQUAST was used for ground truth assessment of the simulated mock (SM) community assemblies by providing the list of 73 FASTA format reference genomes (“-R”). The CPM measure was computed based on the information derived from the results of MetaQUAST [54]. In order to be consistent with the reported values (i.e., N50 length), the CPM measures reported within this article are based on alignments of 500 bp and above, unlike the 1-kb cutoff used in the original work [62]. Prodigal was also used for gene prediction to obtain the number of complete and incomplete genes [61].

### Analysis of contigs assembled from MT data

A list of contigs with no MG depth of coverage together with additional information on these contigs (contig length, annotation, MT depth of coverage) was retrieved using the R workspace image, which is provided as part IMP output (sections “Visualization and reporting” and “Output”). The sequences of these contigs were extracted and subjected to a BLAST search on NCBI to determine their potential origin. Furthermore, contigs with length ≥1 kb, average depth of coverage ≥20 bases, and containing genes encoding known virus/bacteriophage functions were extracted.

### Analysis of subsets of contigs

Subsets of contigs within the HF1 dataset were identified by visual inspection of augmented VizBin maps generated by IMP. Specifically, detailed inspection of contig-level MT to MG depth of coverage ratios was carried out using the R workspace provided as part of IMP output (sections “Visualization and reporting” and “Output”). The alignment information of contigs to isolate genomes provided by MetaQUAST [54] was used to highlight subsets of contigs aligning to genomes of the *Escherichia coli* P12B strain (*E. coli*) and *Collinsella intestinalis* DSM 13280 (*C. intestinalis*).

An additional reference-based analysis of MetaQUAST [54] was carried out for all the human fecal microbiome assemblies (HF1–5) by providing the genomes of *E. coli* P12B and *C. intestinalis* DSM 13280 as reference (flag: “-R”) to assess the recovery fraction of the aforementioned genomes within the different assemblies.

### Computational platforms

IMP and MetAMOS were executed on a Dell R820 machine with 32 Intel(R) Xeon(R) CPU E5-4640 @ 2.40GHz physical computing cores (64 virtual), 1024 TB of DDR3 RAM (32 GB per core) with Debian 7 Wheezy as the operating system. MOCAT, IMP single-omic assemblies, and additional analyses were performed on the Gaia cluster of the University of Luxembourg HPC platform [78].

IMP was executed on the Amazon Web Services (AWS) cloud computing platform using EC2 R3 type (memory optimized) model r3.4xlarge instance with 16 compute cores, 122 GB memory, and 320 GB of storage space running a virtual Amazon Machine Image (AMI) Ubuntu v16.04 operating system.

## Additional files

Additional file 1: Supplementary figures and notes. **Figures S1–S3** and **Notes S1–S2**. Detailed figure legends available within file. (PDF 1047 kb)
Additional file 2: Supplementary tables. **Tables S1–S12.** Detailed table legends available within file. (XLSX 4350 kb)

## Abbreviations

AWS: Amazon Web Services
BAM: Binary Alignment Maps
BG: Biogas
bp: Base pair
CAMI: Critical Assessment of Metagenome Interpretation
cDNA: Complementary DNA
Contigs: Contiguous sequence(s)
HF: Human fecal
IGC: Integrated Gene Catalog
IMP: Integrated Meta-omic Pipeline
INDELs: Insertions and deletions
kb: Kilo base
KEGG: Kyoto Encyclopedia of Genes and Genomes
MetaHIT: Metagenomics of the Human Intestinal Tract
MG: Metagenomic
MT: Metatranscriptomic
NCBI: National Center for Biotechnology Information
NGS: Next-generation sequencing
rRNA: Ribosomal RNA
SM: Simulated mock
SNPs: Single nucleotide polymorphisms
SRA: Sequence read archive
VCF: Variant call format
WW: Wastewater
