# Supplementary material for: IMP: a pipeline for reproducible reference-independent integrated metagenomic and metatranscriptomic analyses
Source: Genome Biol. 2016 Dec 16;17:260. doi: 10.1186/s13059-016-1116-8 (PMC5159968; doi:10.1186/s13059-016-1116-8)
Supplement: Additional file 1: — Supplementary figures and notes. Figures S1–S3 and Notes S1–S2. Detailed figure legends available within file. (PDF 1047 kb) [file 13059_2016_1116_MOESM1_ESM.pdf]

Supplementary information for the article IMP: a reproducible pipeline for reference-independent integrated metagenomic and metatranscriptomic analyses

Shaman Narayanasamy[†1], Yohan Jarosz[†1], Emilie E.L. Muller[1°], Anna Heintz-Buschart[1], Malte Herold[1], Anne Kaysen[1], Cédric C. Laczny[1°], Nicolás Pinel[2°], Patrick May[1], and Paul Wilmes[1*]

[†] Equal contributors

* Correspondence

[1] Luxembourg Centre for Systems Biomedicine, 6 avenue du Swing, University of Luxembourg, L-4367 Belvaux, Luxembourg

[2] Institute of Systems Biology, 401 Terry Avenue North, WA 98109, Seattle, USA.

This document is refered to as Additional file 2 within the main text of the article. It contains Supplementary figures and notes referred within the article. Supplementary tables are available in Additional file 3.

Supplementary figures

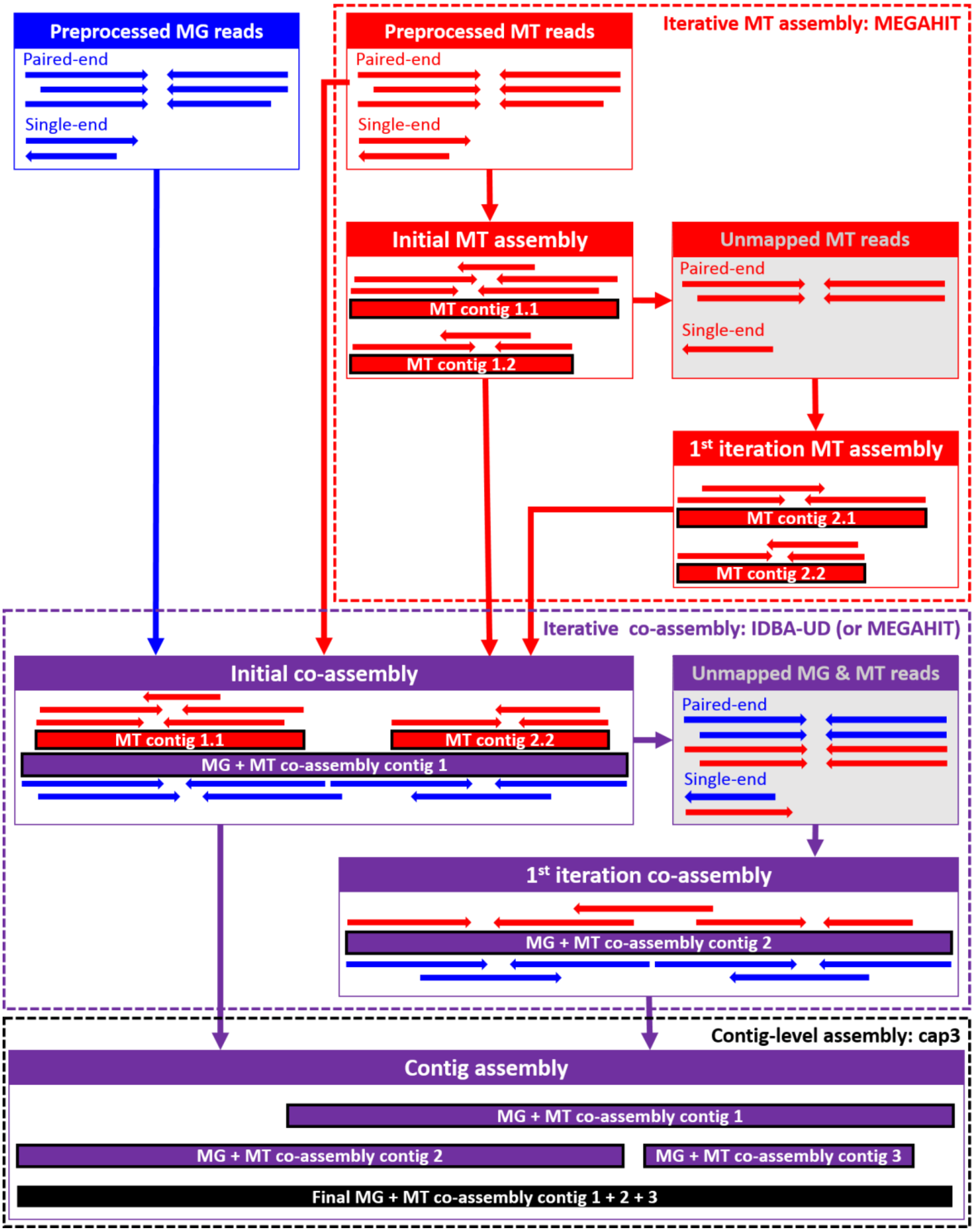

Figure S1. Detailed IMP-based iterative co-assembly procedure. Blue represents metagenomic (MG) data, output and processes. Red represents metatranscriptomic (MT) data, output and processes. Violet represents output and processes involving integrated MG and MT data usage. Dashed-lined boxes represent different iterative assembly steps and contig assemblies, with the respective de novo assembler(s) listed on the top left corner of each box [1–3].

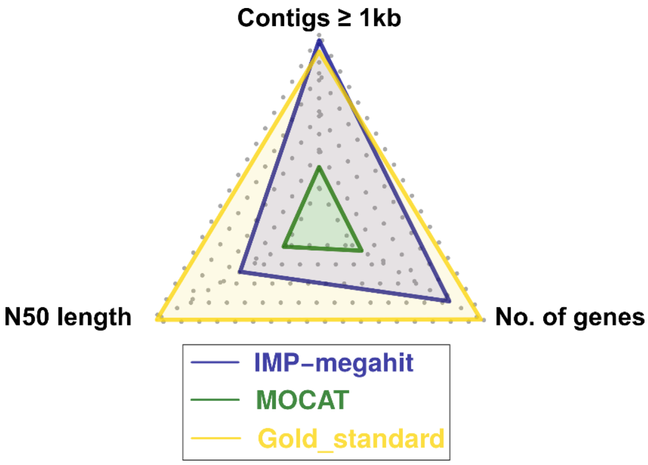

Figure S2. Quality assessment of CAMI medium complexity metagenomic dataset assembly using IMP-megahit and MOCAT compared to the CAMI gold standard assembly (http://www.cami-challenge.org).

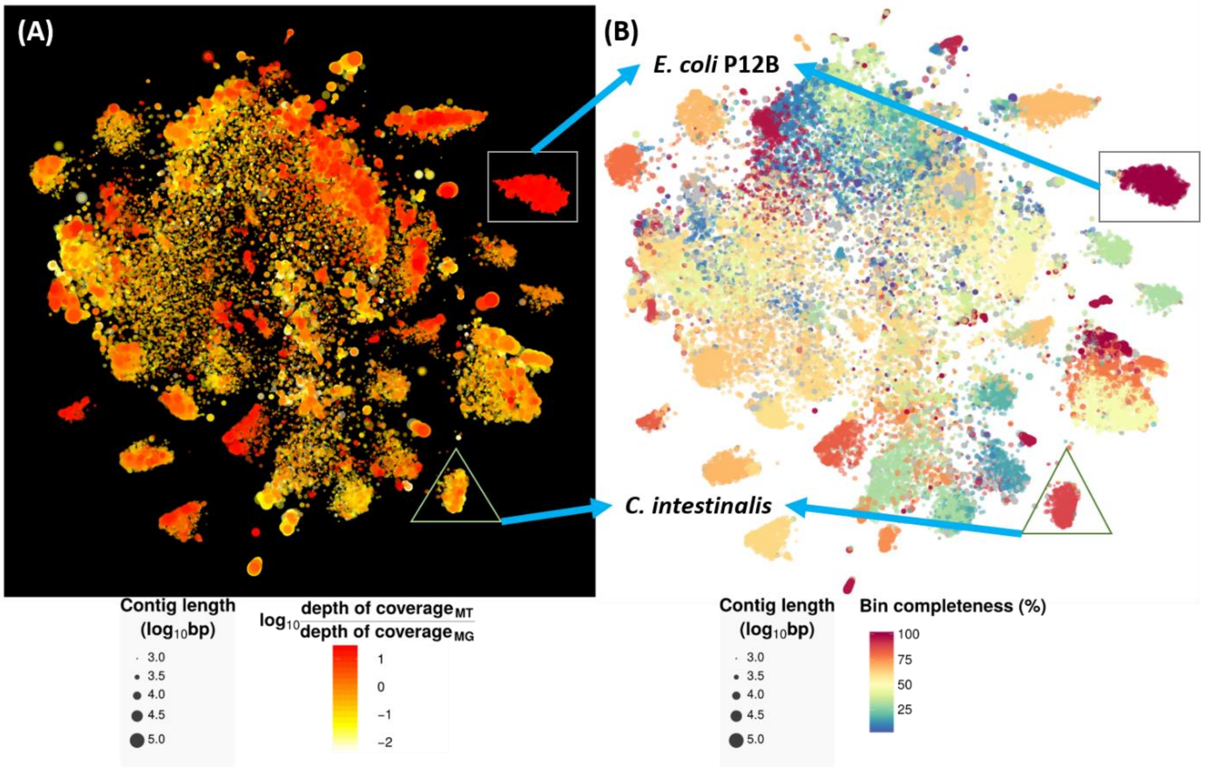

Figure S3. Identification of populations based on metatranscriptional activity. **(A)** Augmented VizBin map [4] showing contig-level metatranscriptomic (MT) to metagenomic (MG) depth of coverage ratios. **(B)** Augmented VizBin map [4] showing completeness of bins identified via automated binning [5]. The squares highlight a subset of contigs that are highly similar to *E. coli* P12B strain while triangles highlight a subset of contigs that are highly similar to *C. intestinalis* DSM 13280 strain.

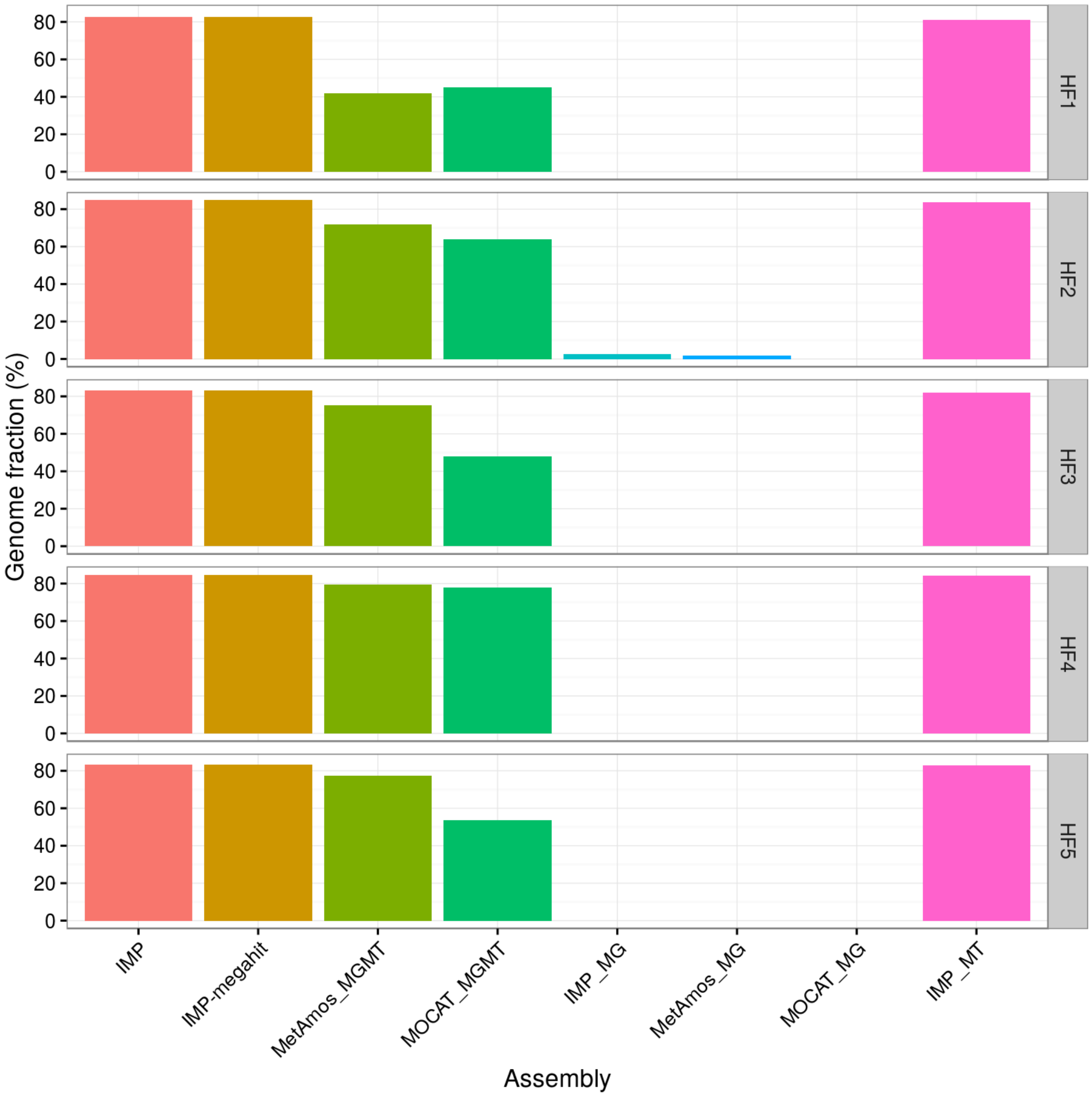

Figure S4. Recovery of *Escherichia coli* P12B genome in the five human fecal microbiome data sets (HF1-5). Bar charts representing the genome fractions in percentage recovered for the aforementioned microbial strain for the different single-omic assemblies (MetAmos_MG [6], MOCAT_MG [7], IMP_MG, IMP_MT) and multi-omic co-assemblies (IMP, IMP-megahit, MetAmos_MGMT, MOCAT_MGMT). For detailed information, refer to Additional files 1: Table S12.

Supplementary notes

Note S1: IMP execution on Amazon cloud computing services (AWS)

IMP ver. 1.4 was launched on the Amazon Web Services (AWS) platform to test IMP on a cloud computing environment. A human fecal sample dataset (HF1) described in the manuscript was used for this test. IMP was launched using the following command:

```
impy -d /mnt/data/db -s IMP --threads 16 --memtotal 120 --memcore 8 run -m
input/X310763260_MG_R1.fq -m input/X310763260_MG_R2.fq -t input/X310763260_MT_R1.fq -t
input/X310763260_MT_R2.fq
```

The total runtime was 824 (13h 44m) minutes. The total storage space required was approximately 97 GB which encompasses all files downloaded and generated from the installation of IMP up to the final analysis of the data using IMP (i.e.: 13GB – db folder, 5.9GB – db.tgz file, 1.5MB – get-pip.py file, 11MB – IMP source code folder, 65GB – imp-output folder and 14GB – input). The operating system image used was: ubuntu/images/hvm-ssd/ubuntu-xenial-16.04-amd64-server-20160721-d83d0782-cb94-46d7-8993-f4ce15d1a484-ami-cf68e0d8.3 (ami-9e6a9ef1).

Note S2: Summary of metagenomic (MG) and metatranscriptomic (MT) data preprocessing

For the ten real datasets described in the article, the IMP preprocessing and filtering procedures for MG data retained 67.78 – 94.54% paired-end reads of which 5.46 – 30.25% were removed due to low quality. For the human fecal MG datasets, 1.0 – 2.22% of read pairs were filtered out because they mapped to the human genome ver. 38 (hg38). For the MT data, a wide range of 5.98 – 90.83% of read pairs were retained of which 5.92 – 33.20% were removed due to low quality. The MT contained also small amounts of human host reads (0.32 – 3.27%). For detailed information about the preprocessing of all datasets, refer to Additional file 3: Table S6.

References

1. Li D, Liu C-M, Luo R, Sadakane K, Lam T-W: **MEGAHIT: an ultra-fast single-node solution**

for large and complex metagenomics assembly via succinct de Bruijn graph. *Bioinformatics* 2015, **31**:1674–1676.

2. Peng Y, Leung HCM, Yiu SM, Chin FYL: **IDBA-UD: a de novo assembler for single-cell and metagenomic sequencing data with highly uneven depth.** *Bioinformatics* 2012, **28**:1420–1428.

3. Huang X, Madan A: **CAP3: A DNA sequence assembly program**. *Genome Res* 1999, **9**:868–877.

4. Laczny CC, Sternal T, Plugaru V, Gawron P, Atashpendar A, Margossian HH, Coronado S, der Maaten L van, Vlassis N, Wilmes P: **VizBin - an application for reference-independent visualization and human-augmented binning of metagenomic data**. *Microbiome* 2015, **3**:1.

5. Wu Y-W, Tang Y-H, Tringe SG, Simmons BA, Singer SW, Metzker M, Dick G, Andersson A, Baker B, Simmons S, Thomas B, Yelton A, Banfield J, Tyson G, Chapman J, Hugenholtz P, Allen E, Ram R, Richardson P, Solovyev V, Rubin E, Rokhsar D, Banfield J, Mackelprang R, Waldrop M, DeAngelis K, David M, Chavarria K, Blazewicz S, Rubin E, et al.: **MaxBin: an automated binning method to recover individual genomes from metagenomes using an expectation-maximization algorithm**. *Microbiome* 2014, **2**:26.

6. Treangen TJ, Koren S, Sommer DD, Liu B, Astrovskaya I, Ondov B, Darling AE, Phillippy AM, Pop M: **MetAMOS: a modular and open source metagenomic assembly and analysis pipeline.** *Genome Biol* 2013, **14**:R2.

7. Kultima JR, Sunagawa S, Li J, Chen W, Chen H, Mende DR, Arumugam M, Pan Q, Liu B, Qin J, Wang J, Bork P: **MOCAT: a metagenomics assembly and gene prediction toolkit.** *PLoS One* 2012, **7**:e47656.
